# Karyotypic evolutions of cancer species in rats during the long latent periods after injection of nitrosourea

**DOI:** 10.1186/s13039-014-0071-x

**Published:** 2014-12-16

**Authors:** Mathew Bloomfield, Amanda McCormack, Daniele Mandrioli, Christian Fiala, C Marcelo Aldaz, Peter Duesberg

**Affiliations:** Department of Molecular and Cell Biology, Donner Laboratory, University of California at Berkeley, Berkeley, CA USA; Department of Environmental Health Sciences, Johns Hopkins Bloomberg School of Public Health, Baltimore, MD USA; Gynmed Ambulatorium, Mariahilferguertel 37, 1150 Vienna, Austria; Department of Molecular Carcinogenesis, M. D. Anderson Cancer Center, Smithville, Texas 78957 USA

**Keywords:** Carcinogens function as aneuploidogens, Persistent non-clonal hyperplasias, Individual clonal karyotypes of cancers, Single-step origins of cancer karyotypes, Karyotype arrays

## Abstract

**Background:**

A century of research has established that cancers arise from tissues exposed to carcinogens only after long latencies of years to decades and have individual clonal karyotypes. Since speciation from known precursors also depends on long latencies and new species also have individual karyotypes, we and others have recently proposed that carcinogenesis is a form of speciation. According to this theory karyotypic evolutions generate new cancer species from normal cells as follows: Carcinogens induce aneuploidy (Figure 1). By unbalancing thousands of genes aneuploidy automatically destabilizes the karyotype and thus catalyzes random karyotypic variations. Selections of variants with proliferative phenotypes form non-clonal hyperplasias with persistently varying karyotypes. Very rare karyotypic variations form new cancer species with individual clonal karyotypes. Despite destabilization by the resulting congenital aneuploidies, cancer karyotypes are stabilized within narrow margins of variation by clonal selections for cancer-specific autonomy. Because all non-cancerous aneuploidies are unstable, all aneusomies of prospective cancers are joined in single-steps, rather than gradually. Since this mechanism is very inefficient, it predicts long latent periods from carcinogens to cancers and individual clonal cancer karyotypes.

**Results:**

Here we have tested the predicted roles of karyotypic evolutions during the time course of carcinogenesis in an established experimental system. In this system injection of nitrosourea induces in female rats non-invasive mammary hyperplasias (“tumors”) after two or more months, and invasive carcinomas after six or more months. Accordingly four specific predictions were tested: (1) Invasive cancers are late and carry individual clonal karyotypes and phenotypes, (2) Persistent hyperplasias carry non-clonal karyotypes, (3) Non-clonal hyperplasias generate clonal cancers spontaneously but rarely, (4) Cancer-karyotypes arise with all individual clonal aneusomies in single-steps. All four predictions were experimentally confirmed.

**Conclusions:**

Our results along with the literature reveal a coherent karyotypic mechanism of carcinogenesis: Carcinogens induce aneuploidy. The inherent instability of aneuploidy automatically catalyzes new karyotypic variations. Aneuploid karyotypes with proliferative phenotypes form varying non-clonal hyperplasias. Rare variations form cancer species with individual clonal karyotypes, which are stabilized by clonal selection for autonomy. The low odds of this mechanism explain the long latencies of carcinogenesis, the individuality and karyotypic clonality of cancers.

## Background

A century of cancer research has established that cancers arise from tissues exposed to carcinogens only after long latencies of years to decades [1-9] and have individual clonal karyotypes [2,10-20].

Several attempts to explain these characteristics of carcinogenesis with the currently prevailing mutation theory of cancer have been unsuccessful. For example, the mutation theory explains the long latencies of years to decades from atomic bomb explosions in 1945 [5,9] or from X-ray therapies of tuberculosis [6] to subsequent cancers by requirements of subsequent mutations. This seems odd, however, in view of the huge loads of mutagenic radiations long before carcinogenesis. Further, a requirement of subsequent mutations does not explain, why even the steady fraction of any population, which has already accumulated or inherited all but one of the hypothetically required mutations, would not have developed cancers without delay. Moreover, the multi-mutation theory of the long latencies of carcinogenesis does not explain the persistent, non-clonal preneoplastic aneuploidies in carcinogen-exposed animals and cancer-free people [10,21-24], specifically in the well-studied survivors of atom bombs [25-27]. (See more examples in, ‘Test-2: do persistent hyperplasias carry varying aneuploidies?’). The mutation theory also does not explain, why all cancers have individual clonal karyotypes and phenotypes, instead of diploid karyotypes with specific sets of mutations [15,20,28]. Since the currently prevailing mutation theory does not answer these questions, the mechanism of carcinogenesis is still a matter of debate [5,29-31]. Accordingly, Brash and Cairns stated in 2009, “mutagenic carcinogens cause just one or two events and that these are then followed by steps that accumulate solely with the passage of time” [32].

Since speciation depends on “the passage of time” and on the generation of new individual karyotypes, several researchers, including us, have recently proposed that carcinogenesis is a form of speciation [16,19,33-37].

**Figure 1:**
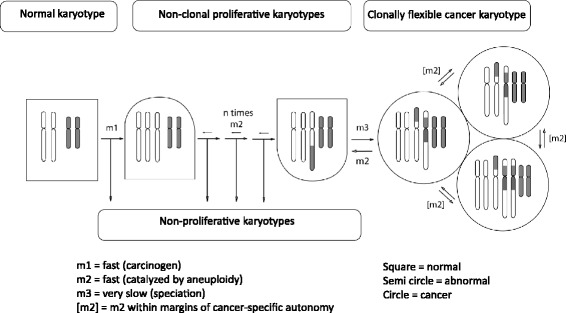
**The speciation theory of cancer.** This theory postulates that carcinogens or spontaneous events induce aneuploidy, namely losses or gains of entire chromosomes or parts of chromosomes at rates, termed m1. By unbalancing thousands of genes aneuploidy automatically destabilizes the karyotype, and thus catalyzes random karyotypic variations at aneuploidy-dependent rates, termed m2. Selections of variants with proliferative phenotypes form non-clonal hyperplasias* with persistently varying karyotypes. Variants without proliferative phenotypes perish. Very rare karyotypic variations form new cancer species with individual clonal karyotypes at very low rates, termed m3. Despite destabilization by their congenital aneuploidies, cancer karyotypes are stabilized within narrow margins of variation by clonal selections for cancer-specific autonomy of growth. Within these margins cancer karyotypes are clonally flexible at aneuploidy-dependent rates, termed [m2]. To generate new cancers all clonal aneusomies of cancers must be joined in single-steps, rather than gradually, because of the inherent instability of non-cancerous or pre-cancerous aneuploidy. Since this mechanism is very inefficient, it predicts long latent periods from carcinogens to cancers and individual clonal cancer karyotypes. *Hyperplasias are defined here according to the Oxford American Dictionary as “The enlargement of an organ or tissue caused by an increase in the reproductive rate of its cells, often as an initial stage in the development of cancer.”

According to this theory karyotypic evolutions generate new autonomous cancer species from normal cells as follows: Carcinogens induce random aneuploidy (Figure 1). By unbalancing thousands of genes aneuploidy automatically destabilizes the karyotype, and thus catalyzes random karyotypic variations [38-40]. Selections of variants with proliferative phenotypes form non-clonal hyperplasias with persistently varying karyotypes. (Hyperplasias are defined according to the Oxford American Dictionary as “The enlargement of an organ or tissue caused by an increase in the reproductive rate of its cells, often as an initial stage in the development of cancer.”) Very rare karyotypic variations form new cancer species with individual clonal karyotypes. Despite destabilization by the resulting congenital aneuploidies, cancer karyotypes are stabilized within narrow margins of variation by clonal selections for cancer-specific autonomy [19] (Figure 1). Cancer-specific karyotypic flexibility is thus a dynamic equilibrium between destabilization by native aneuploidy and stabilization by selection for cancer-specific autonomy [17,19]. Owing to this inherent flexibility the karyotypes of cancer cells can also revert to a non-cancerous aneuploidy. Because all non-cancerous aneuploidies are unstable, all aneusomies of prospective cancer cells are joined in single-steps, rather than gradually. Since this mechanism is very inefficient, it predicts long latent periods from carcinogens to cancers and individual clonal cancer karyotypes.

This theory does not apply to clonal hyperplasias with chromosomally rearranged but balanced karyotypes, as for example the chronic myelogenous leukemia, which is a hyperplasia of terminally differentiating myeloblasts [41,42]. Such clonal hyperplasias might be caused by chromosome rearrangements or by gene mutations. Our theory would, however, apply to the late ‘blast crises’ of myelogenous leukemias, in which the genomically balanced karyotype is displaced by new aneuploid karyotypes forming neoplastic clones of non-differentiating cells [28,41-43].

In the following we test four specific predictions of the speciation theory of cancer according to which rare selections from random karyotypic evolutions determine the time course of carcinogenesis and the individuality of cancers: (1) Invasive cancers are late and carry individual clonal karyotypes and phenotypes, (2) Persistent hyperplasias carry non-clonal aneuploidies, (3) Non-clonal hyperplasias generate clonal cancers spontaneously but rarely, (4) Cancer-karyotypes arise with all clonal aneusomies in single-steps, rather than gradually.

To test these predictions we have analyzed here the karyotypes and phenotypes of six “primary mammary tumors”, which Aldaz et al. isolated from rats about one year (9 to 12 months) after injection of nitrosourea [44]. The six tumors were labeled “RMT” (rat mammary tumor) 47–3, 37–2, 65, 58, 54, 61. Table 1 provides a brief description of cancer-relevant characteristics of these six tumors. In this long-established experimental system a single injection of nitrosourea induces in female rats non-invasive “mammary tumors” or hyperplasias after two or more months, and invasive “tumors” or carcinomas with “abnormal” karyotypes after six or more months [44-48]. Thus, based on the time from the initiating nitrosourea-treatment, the six tumors we studied here could be either preneoplastic non-clonal hyperplasias or cancers with individual clonal karyotypes and phenotypes.

**Table 1:** **Characteristics of Rat Mammary Tumors (RMT)**

RMT*	**Invasive**	**Karyotype**
47-3	Yes	Clonal
37-2	Yes	Clonal
65	No	Non-clonal
58	No	Non-clonal
54	No	Non-clonal
61	No	Non-clonal

*Isolated one year after injection of Nitrosourea. See reference [44] and Results, Test-1: are carcinomas late and carry individual clonal karyotypes and phenotypes? and Test-2: do persistent hyperplasias carry non-clonal aneuploidies? below.

## Results

In the following we describe the four tests of the roles of karyotypic evolutions in carcinogenesis outlined above, based on karyotypic analyses of the six “mammary tumors” isolated by Aldaz et al. from rats about one year after injection of nitrosourea [44], as described in Table 1 and below. In addition we adduce evidence that cancer karyotypes arise with all prospective clonal cancer-specific aneusomies in single-steps, rather than gradually, because non-cancerous aneuploid intermediates are too unstable to support gradual accumulations.

### Test-1: are carcinomas late and carry individual clonal karyotypes and phenotypes?

The speciation theory attributes the long latencies from carcinogen to carcinogenesis and the clonal individuality of cancers to very rare, and thus typically late karyotypic variations, which generate new autonomous cancer-species with individual clonal karyotypes. To test these predictions of the speciation theory, we first analyzed those two of the six RMT “tumors” from Aldaz et al., which were invasive and “metastatic” at the time of isolation a year after nitrosourea, namely RMT 47–3 and 37–2 [44] (Table 1). Since these two “tumors” were metastatic and had been transplanted to isogenic rats several times prior to isolation, they were probably carcinomas [44,48]. The hyphenated numbers indicate transplantations of primary tumors.

**Figure 2:**
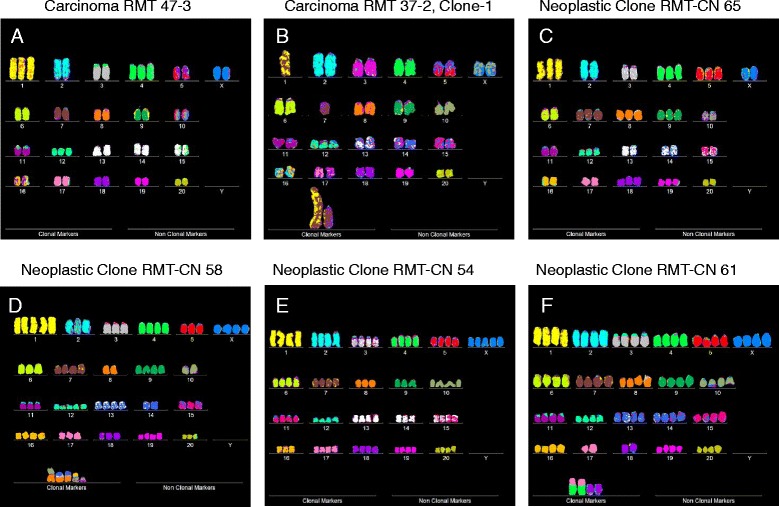
**Karyotypes of two invasive rat mammary carcinomas and of four neoplastic clones evolved from non-clonal hyperplasias in vitro.** The karyotypes of the two rat mammary carcinomas RMT 47–3 **(A)** and 37–2 **(B)** were prepared from short-term cultures of the two carcinomas treated with colcemid for several hours following published procedures [17,49]. Prior to karyotyping the metaphase chromosomes were hybridized with DNA probes carrying chromosome-specific fluorescent colors (Methods). The chromosomes were then arranged into conventional karyotypes with a computer-assisted Zeiss microscope [17,49]. The karyotypes of the four clonal neoplasias RMT-CN 65 **(C),** RMT-CN 58 **(D)**, RMT-CN 54 **(E)** and RMT-CN 61 **(F),** which had evolved spontaneously from cultures of preneoplastic hyperplasias propagated in vitro, were prepared as described for the two carcinomas.

Representative karyotypes of RMT 47–3 and 37–2, shown in Figure 2A and B, indicate that the two carcinomas have individual karyotypes.

To determine whether these karyotypes are clonal, as predicted by the speciation theory (Figure 1), multiple karyotypes of the same cancer must be compared. For this purpose we have used 3-dimensional arrays of 20 karyotypes, which list chromosome numbers on the x-axis, chromosome copy numbers on the y-axis and the numbers of karyotypes arrayed on the z-axis. This method and the preparation of 20 individual karyotypes of the invasive “tumors” RMT 47–3 and 37–2 from color-coded metaphase chromosomes followed published procedures [17,24,49].

**Figure 3:**
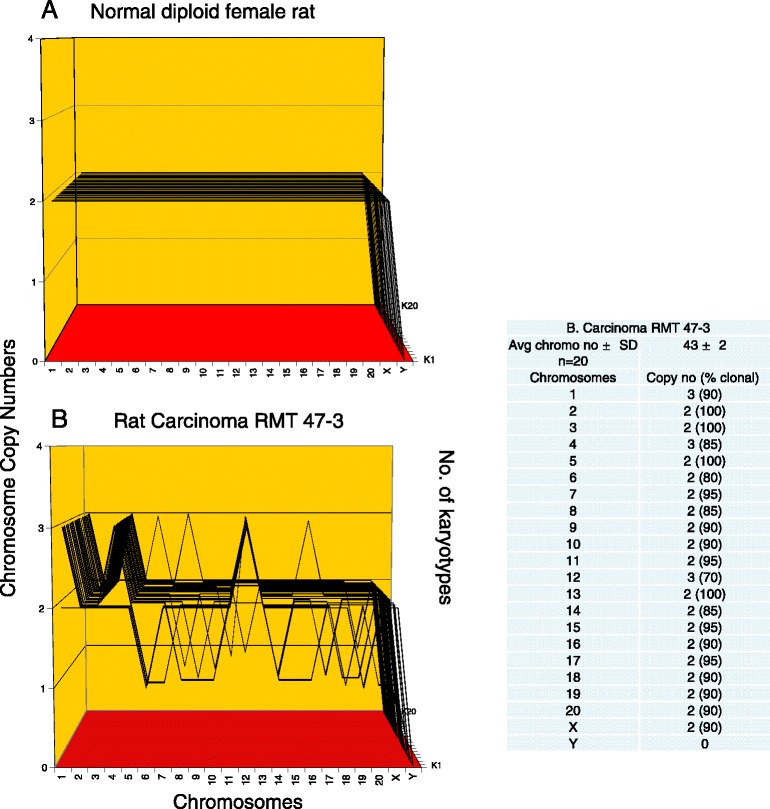
**Karyotype arrays of a normal female rat and of the rat mammary carcinoma RMT 47–3.** Karyotype arrays are three-dimensional tables, which list the chromosome numbers of individual karyotypes on the x-axis, the copy numbers of each chromosome on the y-axis, and the number of karyotypes arrayed on the z-axis. In such arrays the karyotypes with identical, clonal chromosome copy numbers form parallel lines (see text). **(A)** An array of 20 karyotypes of a normal, diploid female rat. **(B)** An array of 20 karyotypes of the rat mammary carcinoma RMT 47–3. The karyotype array and the attached table show that the numbers of RMT 47–3 chromosomes per cell are quasi-clonal, averaging 43 ± 2. The data also show that the copy numbers of RMT 47–3 chromosomes are 70-100% clonal. The minority of non-clonal chromosomes oscillated within narrow margins of ±1 of clonal values, as predicted by the speciation theory (Background and Figure 1).

Because all karyotypes with identical and thus clonal chromosome copy numbers form parallel lines in karyotype arrays, clonality and individuality can be recognized at a glance - much like individual signatures. As an example we show in Figure 3A the 100%-clonal karyotype array of a normal female rat.

The karyotype array of the invasive tumor RMT 47–3 and the underlying quasi-clonal chromosome copy numbers of the 20 RMT 47–3 karyotypes are shown in Figure 3B and in the corresponding table. The table indicates the average chromosome number per RMT 47–3 cell was 43 ± 2. By contrast, the normal chromosome number of rats is 42. The array shows at a glance that the chromosome copy numbers of the invasive RMT 47–3 tumor are highly, 70-100% clonal. The minority of 0-30% of non-clonal chromosome copy numbers oscillated ±1 around clonal averages. The resulting clonal heterogeneity is consistent with the dynamic equilibrium between the inherent instability of the congenital aneuploidy of cancers and the clonal selection for cancer-specific autonomy that stabilizes the karyotypes of cancers within narrow margins (see above, Figure 1, Background and references [17,19]). The invasive tumor RMT 47–3 thus has a near-clonal karyotype, as predicted for invasive cancers by the speciation theory.

With regard to its individuality the karyotype-array of RMT 47–3 was first compared to that of the normal rat and then to further rat cancers (below). As can be seen in Figure 3A and B, the karyotype of RMT 47–3 differs from that of the normal female rat in three clonal, numerical chromosome alterations also termed aneusomies, namely trisomies 1, 4 and 12 (see also Figure 2A), and in a low clonal percentage of heterogeneity. This result extends and confirms a previous karyotypic analysis of RMT 47–3 by Aldaz et al. from 1992 [44] and thus also confirms the predicted clonal stability of this carcinoma from the time of its isolation to the present re-analysis. It is shown below that the RMT 47–3 karyotype also differs from those of the five other neoplastic RMT clones described below.

**Figure 4:**
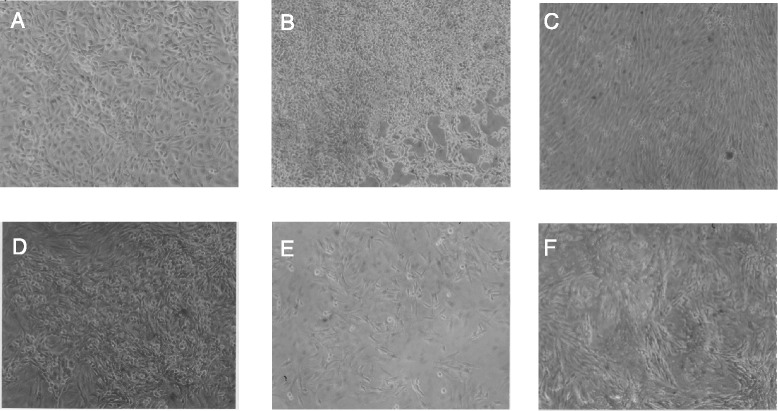
**Cellular morphologies of two rat mammary carcinomas and of four neoplastic clones evolved from rat hyperplasias in vitro.** Cells of the rat mammary carcinomas RMT 47–3 and 37–2 and of four clonal neoplasias derived from mammary hyperlasias termed RMT-CN 65, RMT-CN 58, RMT-CN 54 and RMT-CN 61 were photographed in cell culture dishes at a magnification of 100x with a phase-contrast microscope. The following individualities were observed: **(A)** A dense monolayer of polygonal cells of the RMT 47–3 carcinoma, **(B)** Three-dimensional colonies of round and refractile cells of the carcinoma RMT 37–2 clone 1, **(C)** A dense monolayer of fusiform cells of the clonal neoplasia RMT-CN 65, **(D)** A three-dimensional multilayer of pleomorphic-round cells of the clonal neoplasia RMT-CN 58, **(E)** A relatively flat monolayer of fusiform, triangular cells of the clonal neoplasia RMT-CN 54, **(F)** A three-dimensional cell layer of elongated refractile cells of the clonal neoplasia RMT-CN 61.

The speciation theory further predicts that the clonal karyotype of RMT 47–3 encodes a clonal and thus a uniform cellular phenotype. As shown in Figure 4A, the morphological uniformity of a population of RMT 47–3 cells confirms this prediction. The cellular morphology of RMT 47–3 was also individual based on comparisons with those of the five other clonal rat mammary carcinomas and neoplastic clones described below. (No normal cell control is shown, because the exact cell of origin is not known.) Thus RMT 47–3 has an individual clonal karyotype and phenotype.

**Figure 5:**
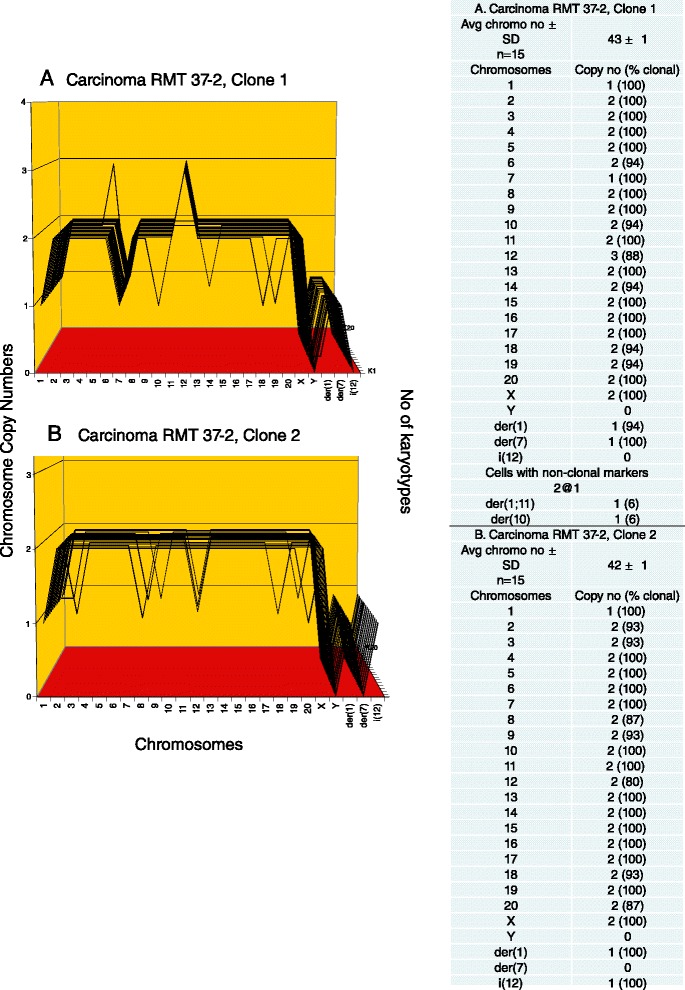
**Karyotype arrays of the rat mammary carcinoma RMT 37–2.** The mammary carcinoma RMT 37–2 consisted of two clones with distinct but related karyotype arrays, clones 1 **(A)** and 2 **(B).** As shown in the attached table the numbers of chromosomes per RMT 37–2 cell are quasi-clonal, averaging of 43 ± 1 and 42 ± 1 respectively. The copy numbers of the normal and marker chromosomes of the two clones are between 80 and 100% clonal. Both RMT 37–2 clones shared a monosomy of the normal chromosome 1 and one clonal, oversized chromosome 1-derived marker chromosome, in which about half of the normal chromosome 1 was duplicated (See Figure 2B). On the other hand, clone-1 differed from clone-2 in a clone-1-specific monosomy 7, a trisomy 12 and a marker chromosome der(7). And clone 2 differed from clone 1 in a marker chromosome of its own. It follows that the two clones either derived from a common ancestor or from each other. The individuality of the two RMT 37–2 karyotypes is evident from comparisons with the karyotype arrays of RMT 47–3 and the normal rat shown in Figure 3.

Next, we analyzed the second invasive and metastatic rat mammary tumor, and thus probable carcinoma RMT 37–2 for karyotypic clonality and individuality. As shown by the two karyotype-arrays in Figure 5A and B and the corresponding tables, RMT 37–2 consisted of two related but distinct clones. Both clone-1 and −2 were near diploid, containing clonal averages of 43 and 42 chromosomes per cell respectively. The copy numbers of these chromosomes were between 87 and 100% clonal. Both RMT 37–2 clones shared a monosomy of the normal chromosome 1 and one clonal, oversized chromosome 1-derived marker chromosome, in which about half of the normal chromosome 1 was duplicated (See Figure 2B). On the other hand, clone-1 differed from clone-2 in a clone-1-specific monosomy 7, a trisomy 12 and a clone-1-specific marker chromosome. Clone 2 also contained a clone-2-specific marker chromosome. It follows that the two clones either derived from a common ancestor or from each other.

The finding of a distinctive chromosome 1-derived marker chromosome in RMT 37–2 again confirmed and extended a prior observation of this marker by Aldaz et al. [44]. Taken together the two carcinomas confirm the idea that, despite inherent flexibility, the karyotypes of cancers are stable, but flexible clones (Figure 1).

The clonality of the two related RMT 37–2 clones predicted again that they should encode uniform cellular phenotypes. It is shown in Figure 4B that the uniform morphology of the cells of RMT 37–2 clone-1 confirmed this prediction.

Although the exact times of the origins of the two carcinomas after the initiating nitrosourea injection cannot be determined based on the available data, we can estimate that the latent periods from nitrosourea to the origins of the two clonal carcinomas were between 6 and 12 months. This follows, because (a) the two carcinomas were isolated about one year after nitrosourea, and (b) no invasive cancers have been observed in this system prior to six months after nitrosourea by others, and us [44,47].

#### Test-1-specific conclusions

1. The karyotypic and phenotypic individualities and clonalities of the invasive tumors or carcinomas confirm the prediction of the speciation theory that the cancers RMT 47–3 and RMT 37–2 originated from individual clonogenic karyotypes, rather than from common mutations, as for example from a common mutant *ras* gene (Background and Discussion).
2. The long, although not accurate latent periods of 6 to 12 months from nitrosourea to the two carcinomas confirm the inevitably inefficient and thus slow chromosomal mechanism of carcinogenesis via random karyotypic variations predicted by the speciation theory.

### Test-2: do persistent hyperplasias carry non-clonal aneuploidies?

Next, we tested the prediction of the speciation theory that persistent hyperplasias (defined in Background and Figure 1) carry varying non-clonal aneuploidies with proliferative phenotypes. For this purpose we have karyotyped the four “tumors” isolated by Aldaz et al. namely RMT 65, 58, 54 and 61, which appeared to fit the definition of a hyperplasia, because they were non-invasive a year after nitrosourea. In the following we show that all four non-invasive tumors analyzed here contained indeed non-clonal aneuploid karyotypes.

A karyotype analysis of 20 cells of the non-invasive “tumor” RMT 65 is shown in Table 2. As can be seen in this table, 10 of 20 (50%) cells of RMT 65 contained non-clonal aneuploid, near-tetraploid karyotypes. The remaining 10 RMT 65 cells contained normal tetraploid karyotypes (not shown). We remind the reader that tetraploidy is a minor, but normal variant of the diploid karyotype. We conclude that the non-invasive RMT 65 tumor is a 50%-aneuploid, non-clonal hyperplasia, consistent with our theory. The 50% of normal tetraploid RMT 65 cells could either represent a “regenerative hyperplasia” [50] or could be entirely normal cells.

**Table 2:** **Non-clonal hyperplasia RMT 65: 10 aneuploid cells in a sample of 20**

Karyotypes	1	2	3	4	5	6	7	8	9	10
Total no. of chromosomes	85	79	84	81	76	70	83	86	84	84
Chromosomes										
1	3	3	4	4	4	4	4	4	4	4
2	3	3	3	3	5	4	4	4	4	4
3	4	4	4	3	4	3	3	4	4	4
4	4	3	5	4	4	3	4	6	4	4
5	4	4	3	3	4	3	4	4	4	4
6	4	4	4	4	4	3	4	4	4	4
7	4	4	4	4	4	3	4	3	4	4
8	4	4	4	4	3	3	4	4	4	4
9	4	4	5	4	2	3	4	5	4	4
10	4	4	4	4	4	4	4	4	4	4
11	4	4	4	4	3	3	3	4	4	4
12	4	3	5	4	4	2	4	5	4	4
13	4	3	4	4	4	3	4	4	4	4
14	4	3	3	4	4	3	4	4	4	4
15	4	4	4	4	4	3	4	3	4	4
16	4	4	4	4	3	4	4	4	3	4
17	4	4	4	4	4	4	4	4	4	4
18	4	4	4	4	4	3	4	4	4	4
19	4	4	4	4	3	4	4	4	4	3
20	4	3	4	4	3	4	4	4	4	4
X	4	4	4	4	2	4	4	4	4	4
Y	0	0	0	0	0	0	0	0	0	0
der(19;8)	0	0	0	0	0	0	0	0	0	1
der (1;2)	1	1	0	0	0	0	0	0	0	0
der (1)	2	0	0	0	0	0	0	0	0	0
der (16;11)	0	0	0	0	0	0	0	0	1	0
der (3;11)	0	0	0	0	0	0	1	0	0	0
der (1;13)	0	1	0	0	0	0	0	0	0	0

As shown in Table 3, 11 of 20 cells of the non-invasive “tumor” **RMT 58** contained non-clonal aneuploid karyotypes. The remaining nine cells contained normal diploid karyotypes (not shown). It follows that the non-invasive “tumor” RMT 58 was a 55%-aneuploid, non-clonal hyperplasia.

**Table 3:** **Non-clonal hyperplasia RMT 58: 11 aneuploid cells in a sample of 20**

Karyotypes	1	2	3	4	5	6	7	8	9	10	11
Total no. of chromosomes	35	42	32	32	32	35	41	38	39	41	41
Chromosomes											
1	1	3	2	2	2	2	2	2	2	2	2
2	2	2	1	1	1	1	3	2	2	2	2
3	2	2	2	2	2	1	2	1	2	2	2
4	2	2	2	2	2	2	2	2	2	2	2
5	2	2	1	1	1	1	2	2	1	2	2
6	2	2	2	2	1	2	2	1	2	2	2
7	1	2	2	2	2	2	2	2	2	2	2
8	1	2	0	1	2	1	1	2	2	2	2
9	2	2	1	2	0	2	2	2	1	2	2
10	1	2	2	1	2	1	2	2	2	2	2
11	1	2	2	1	2	2	2	2	2	2	2
12	2	2	1	3	1	2	2	2	2	2	2
13	2	2	2	3	2	2	2	2	1	2	2
14	2	1	1	2	2	2	2	2	2	2	2
15	2	2	0	1	1	2	1	2	2	2	2
16	2	2	1	0	2	2	2	2	2	2	2
17	2	2	2	2	1	2	2	2	2	1	1
18	1	2	2	0	2	1	2	1	2	2	2
19	2	2	2	1	2	2	2	2	2	2	2
20	1	2	2	2	1	1	2	2	2	2	2
X	2	2	2	1	1	2	2	1	2	2	2
Y	0	0	0	0	0	0	0	0	0	0	0

As shown in Table 4, 17 of 20 cells of the non-invasive “tumor” **RMT 54** contained non-clonal aneuploid karyotypes. The remaining three cells were normal diploid cells (not shown). Thus RMT 54 was an 85%-aneuploid, non-clonal hyperplasia.

**Table 4:** **Non-clonal hyperplasia RMT 54: 17 aneuploid cells in a sample of 20**

Karyotypes	1	2	3	4	5	6	7	8	9	10	11	12	13	14	15	16	17
Total no. of chromosomes	43	35	42	42	42	42	42	42	42	42	42	42	42	42	41	42	42
Chromosomes																	
1	3	1	2	2	2	2	2	2	2	2	2	2	2	2	2	2	2
2	2	2	2	2	2	2	2	2	2	2	2	2	2	2	2	2	2
3	1	1	2	2	2	2	2	2	2	2	2	2	2	2	2	2	2
4	2	1	1	1	1	1	1	2	2	2	2	2	2	2	2	2	2
5	2	2	2	2	2	2	2	3	2	2	2	2	2	2	2	2	2
6	2	1	2	2	2	2	2	2	2	2	2	2	2	2	2	2	2
7	2	2	2	2	2	2	2	2	2	2	2	2	2	2	2	2	2
8	2	2	2	2	2	2	2	2	2	2	2	2	2	2	2	2	2
9	2	2	2	2	2	2	2	2	2	2	2	2	2	2	2	2	2
10	2	2	1	2	2	2	2	2	1	2	2	2	2	2	2	2	2
11	2	1	2	1	2	2	2	2	2	1	1	2	2	2	2	2	2
12	2	1	2	2	2	1	2	2	1	2	2	2	2	2	2	2	2
13	2	1	3	3	2	2	2	2	2	2	3	1	2	2	2	2	2
14	2	2	2	3	3	2	2	2	2	2	3	2	3	2	2	2	2
15	2	1	1	1	1	2	2	2	2	2	1	2	1	2	2	2	2
16	2	2	2	2	2	2	2	2	2	2	2	2	2	2	2	2	2
17	2	2	2	2	2	2	2	2	2	2	2	2	2	2	2	2	2
18	2	2	2	2	2	2	1	2	2	2	2	2	2	1	2	2	2
19	1	1	2	2	1	2	2	2	2	2	2	1	2	1	1	1	2
20	2	2	2	2	2	2	2	2	2	2	2	2	2	2	2	2	2
X	1	2	2	2	2	2	2	1	2	1	2	2	2	1	2	2	1
Y	0	0	0	0	0	0	0	0	0	0	0	0	0	0	0	0	0
der(3)	1	1	1	1	1	1	1	0	0	0	0	1	0	0	0	0	0
der(8)	1	0	0	0	1	0	0	0	0	0	0	1	0	1	0	1	0
der(2)	1	1	1	0	0	0	0	0	1	1	0	0	0	2	0	0	1
der(13)	0	0	0	0	0	0	0	0	0	1	0	0	0	0	0	0	0
der(5)	0	0	0	0	0	1	1	0	0	0	0	0	0	0	0	0	0
der(9)	0	0	0	0	0	0	0	0	1	0	0	0	0	0	0	0	0

Finally, we show in Table 5 that 12 of 20 cells of the non-invasive “tumor” **RMT 61** contained non-clonal aneuploid karyotypes. Five of these cells consisted of near-diploid aneuploid, and seven of near-tetraploid aneuploid karyotypes. The remaining eight cells were five normal diploid and three normal tetraploid cells (not shown). Thus RMT 61 was a 60%-aneuploid, non-clonal hyperplasia.

**Table 5:** **Non-clonal hyperplasia RMT 61: 12 aneuploid cells in a sample of 20**

Karyotypes	1	2	3	4	5	6	7	8	9	10	11	12
Total no. of chromosomes	45	40	43	43	43	82	82	84	82	85	85	86
Chromosomes												
1	2	2	2	2	2	4	4	4	4	4	4	4
2	3	2	2	2	2	3	4	4	4	4	4	4
3	2	2	2	2	2	4	4	4	4	4	4	4
4	2	2	2	2	2	4	4	4	4	4	4	4
5	2	2	2	2	2	4	4	4	4	4	4	4
6	2	2	2	2	2	4	4	4	4	4	4	4
7	2	2	2	2	2	4	3	4	4	4	4	4
8	2	2	2	2	2	4	4	4	4	4	4	4
9	2	2	2	2	2	4	4	4	4	4	4	4
10	2	2	2	2	2	4	4	4	4	4	4	4
11	2	2	2	2	2	3	4	4	4	4	4	4
12	2	2	2	2	2	4	3	5	4	4	4	4
13	3	2	2	2	2	4	4	4	4	4	4	4
14	2	2	2	2	2	4	4	4	4	4	4	4
15	2	1	2	2	2	4	4	4	3	5	5	4
16	3	1	2	2	2	4	4	4	4	4	4	4
17	2	2	2	2	2	4	4	4	4	4	4	4
18	2	2	2	2	2	4	4	4	4	4	4	4
19	2	2	2	2	2	4	4	4	3	4	4	4
20	2	2	2	2	2	4	4	4	4	4	4	4
X	2	2	2	2	2	4	4	3	4	4	4	6
Y	0	0	0	0	0	0	0	0	0	0	0	0
der(?)	0	0	1	0	0	0	0	0	0	0	0	0
broken X	0	0	0	1	0	0	0	0	0	0	0	0
der (7 ?)	0	0	0	0	1	0	0	0	0	0	0	0

In sum, the four non-invasive, nitrosourea-induced rat tumors analyzed here had 50-85%-aneuploid, non-clonal karyotypes. These results confirm and extend the results of Goepfert et al., who found that the aneuploid fractions of cells of the “preneoplastic phase” of nitrosourea-treated rats “ranged from 35 up to 82%” [47].

In the following we list parallel rat-, other animal- and human preneoplastic systems, in which persistent proliferative hyperplasias with non-clonal aneuploidies have been observed previously (see also Background):

1. Studying rat tracheal primary cells 5–6 weeks after a single treatment with nitrosoguanidine Barrett et al. observed in 1988 that selection for “enhanced growth” coincided with non-clonal, “abnormal karyotypes” [51]. Non-clonal aneuploid “hyperplasias” were also observed (a) in rats treated with nitrosamine for 10 weeks in 2009 [52], (b) in rats treated “early”, from 8 to 57 days with methylcholanthrene in 1975 [53,54], and (c) in rats treated “early”, from 2.5 to 6 months after treatments with azo-dyes and radiation in 1960 and 1963 [55,56] and with butter yellow in 1957 [57].
2. Enhanced or hyperplastic growth segregating with non-clonal aneuploidy was also observed (a) by Levan in spontaneously transforming mouse cells in vitro in 1958 [10], (b) and by Hauschka in carcinogen-treated mice, “One of the first precancerous reactions was the appearance of numerous mitotic abnormalities with a frequency of up to 60 percent” [21] and (c) by Nowell in cells of irradiated mice, “… with extensive chromosome changes [that] may persist in the hematopoietic tissues for long periods after irradiation without leukemia arising” [22].
3. Preneoplastic non-clonal aneuploid hyperlasias have also been observed by Awa et al. in cancer-free atom bomb survivors after 1945 as “Persistent chromosome aberrations in the somatic cells of A-bomb survivors, Hiroshima and Nagasaki”, in 1974, 1991 and 1997 [25-27].
4. Preneoplastic non-clonal aneuploidy was also observed (a) in irradiated human T-cells prior to neoplastic transformation [58], (b) in aneuploid hyperplasia derived from irradiated human lymphocytes in 1996 [59], and (c) in 1998 as “A typical feature of chromosomal instability in primary human G0-lymphocytes exposed to gamma-irradiation … [causing] the appearance of novel aberrations in the clonal progeny of the irradiated cell, many generations after the exposure” [23].
5. Preneoplastic, non-clonal aneuploidy was also found in precursors of spontaneously transformed Chinese hamster cells [60], and in our lab in preneoplastic Chinese hamster cells treated with nitrosourea [61].

In sum, all of these prior studies confirm the occurrence of non-clonal, persistent hyperplasias prior to the origins of immortal neoplastic clones or clonal cancers, but a coherent theory did not emerge. For example, Barrett et al. closed their study (listed as example (1) above) on the non-clonal preneoplastic hyperplasias of rat cells treated with nitrosoguanidine, “These aneuploid cells clearly have a selective advantage in this system but the reasons for this are unclear” [51].

#### Test-2-specific conclusion

We conclude that the 1-to-1 correlations between non-clonal, preneoplastic aneuploidy and hyperplasia observed in rats here and in rats, mice, hamsters and humans previously directly support the prediction of the speciation theory that non-clonal hyperplasias with varying aneuploidies may persist over years until they are displaced by clonal immortal cancers or perish (Figure 1).

Nevertheless, the significance of the persistent preneoplastic aneuploidies for carcinogenesis observed here in rats and in the different systems listed above depends on proof that these aneuploidies are precursors of the karyotypes of clonal cancers. To test this prediction, we have investigated next, whether the four aneuploid rat hyperplasias studied here (Table 1) generate new clonal neoplasias spontaneously.

This seemed plausible; because we found recently that non-clonal hyperplasias induced in human skin cells by treatments with artificially over-expressed hypothetical cancer genes persisted over 100 generations in vitro prior to the formation of immortal neoplastic clones. New immortal and tumorigenic clones only evolved after over 100 aneuploid cell generations in vitro [24,62].

### Test-3: do non-clonal hyperlasias generate clonal cancers spontaneously but rarely?

The speciation theory predicts that the inherently variable aneuploidies of preneoplastic hyperplasias function as precursors for the stochastic evolution of cancer karyotypes (See Figure 1). To test this prediction we have analyzed cultures of the four non-clonal and noninvasive hyperplasias RMT 65, 58, 54 and 61 (Tables 1, 2, 3, 4 and 5) in vitro, allowing time for the spontaneous evolution of new neoplastic clones.

Surprisingly all four non-clonal and non-invasive rat hyperlasias evolved new three-dimensionally growing foci of clonal neoplasias (CN) within a few months of propagation in vitro. Hence, we called these clones RMT-CN 65, 58, 54 and 61. The relatively fast evolutions of new neoplastic clones within a few months in culture may be due to the high mitotic rates of the hyperplastic rat cells in culture. By contrast, growth in vivo must be more restrained or else the hyperplasias would outgrow the rats within much less than a year. Recently we have also observed similar high growth rates in aneuploid hyperplastic human cells in cell culture [24].

**Figure 6:**
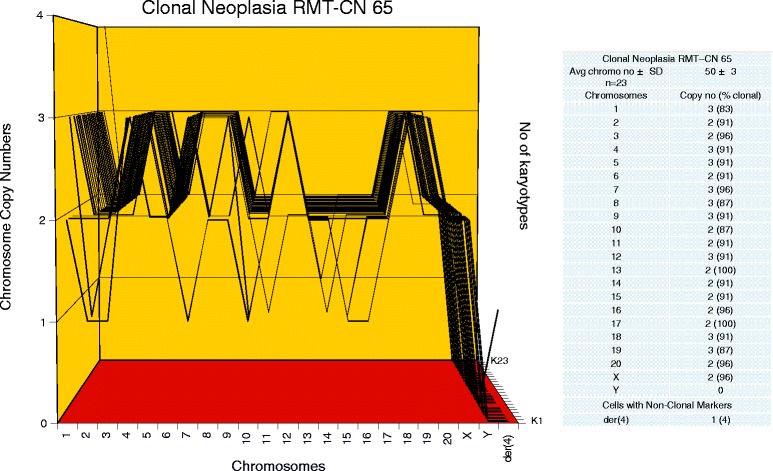
**Karyotype array of the clonal neoplasia RMT-CN 65, which evolved from a non-clonal rat mammary hyperplasia.** The karyotype array of the clonal neoplasia RMT-CN 65 was prepared from a three-dimensionally growing focal colony that had evolved from a culture of the non-clonal aneuploid mammary hyperplasia RMT 65 within several months in culture (Table 2). The karyotype array and the attached table show that the numbers of RMT-CN 65 chromosomes per cell were quasi-clonal, averaging 50 ± 3. The data also show that the chromosome copy numbers were 83-100%-clonal, and that the RMT-CN 65 karyotype is individually distinct from those shown in Figures 3 and 5.

**Figure 7:**
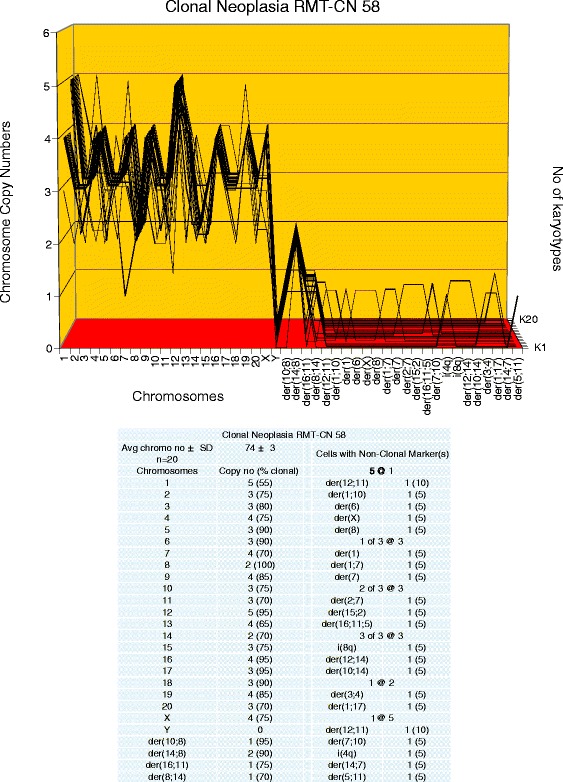
**Karyotype array of the clonal neoplasia RMT-CN 58, which evolved from a non-clonal rat mammary hyperplasia.** The karyotype array of the neoplastic clone RMT-CN 58 was prepared from a three-dimensionally growing focal colony that had evolved from a culture of the non-clonal aneuploid mammary hyperplasia RMT 58 within several months in culture (Table 3). The karyotype array and the attached table show that the numbers of RMT-CN 58 chromosomes per cell were quasi-clonal, averaging 74 ± 3. The data also show that the copy numbers of the normal and of four clone-specific marker chromosomes of RMT-CN 58 were 55-100%-clonal. The individuality of the RMT-CN 58 karyotype is again evident from comparisons with the karyotype arrays of the carcinomas RMT 47–3 and 37–2 and the neoplastic clone RMT-CN 65 shown in Figures 3, 5 and 6. The RMT-CN 58 clone stands out by a high level of ongoing karyotypic variations, generating karyotypes with up to five new non-clonal aneusomies per cell.

**Figure 8:**
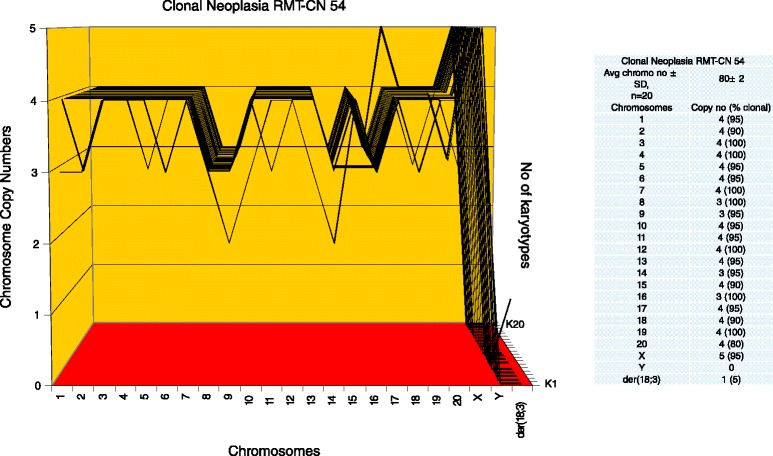
**Karyotype array of the clonal neoplasia RMT-CN 54, which evolved from a non-clonal rat mammary hyperplasia.** This karyotype array of the neoplastic clone RMT-CN 54 was prepared from a three-dimensionally growing colony that had evolved from a culture of the non-clonal aneuploid mammary hyperplasia RMT 54 within several months in culture (Table 4). The karyotype array and the attached table show that the RMT-CN 54 clone contained per cell a quasi-clonal, near-tetraploid average number of 80 ± 2 chromosomes. The data also show that the chromosome copy numbers of the clone were 80-100%-clonal, based on a normal tetraploid karyotype. The individuality of the RMT-CN 54 karyotype array is again evident from comparisons with the karyotype arrays of the carcinomas RMT 47–3 and 37–2 and of the clonal neoplasias RMT-CN 65 and 58 shown in Figures 3, 5, 6 and 7.

**Figure 9:**
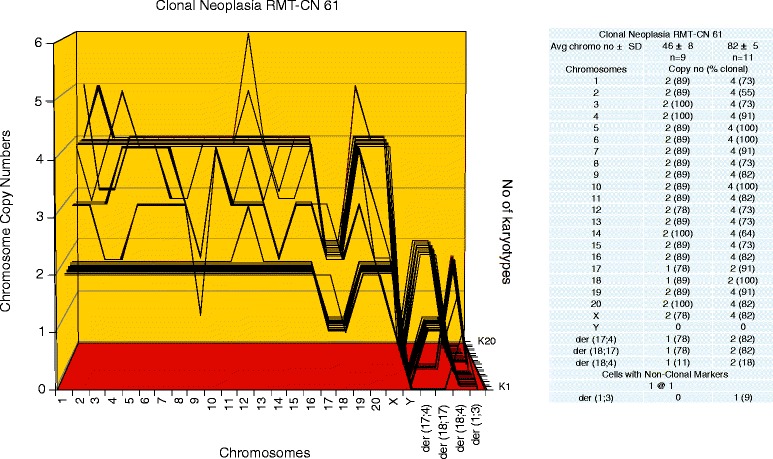
**Karyotype arrays of two related clonal neoplasias RMT-CN 61, which evolved from a non-clonal rat mammary hyperplasia.** The two related karyotype arrays shown here on the same plot were prepared from three-dimensionally growing neoplastic foci termed RMT-CN 61, which had evolved from a culture of the non-clonal aneuploid mammary hyperplasia RMT 61 within several months (Table 5). A glance at the two karyotype-arrays of the RMT-CN 61 foci shows two closely related clonal karyotypes, one near-diploid and the other near-tetraploid. The two RMT-CN 61 clones contained quasi-clonal chromosome numbers, which averaged 46 ± 8 and 82 ± 5 per cell, respectively. The copy numbers of their normal chromosomes and their common and individual marker chromosomes were 73-100%-clonal. The individuality of the two sister clones is evident from comparisons of their karyotype arrays with the five individual karyotype arrays of the two rat carcinomas and the three neoplastic rat clones shown in Figures 3, 5, 6, 7 and 8.

To determine, whether the karyotypes of these new neoplastic clones were clonal, arrays of 20 karyotypes were prepared as described above (Test-1: are carcinomas late and carry individual clonal karyotypes and phenotypes?). Representative karyotypes of the four new neoplastic clones RMT-CN 65, 58, 54 and 61, are shown in Figure 2C,D,E and F respectively. As can be seen in Figures 6, 7, 8 and 9, each of the four new neoplastic rat clones, RMT-CN 65, 58, 54 and 61 had an individual, quasi-clonal karyotype, as is typical of cancer cells. The karyotype of RMT-CN 61 consisted of a near-diploid and a near-tetraploid clone. Since a pair of identical marker chromosomes in primary cancers is extremely rare, the presence of three such markers in the tetraploid variant proves that it derived from the diploid variant by a form of endomitosis.

As expected from the karyotypic clonality of the RMT-CN 65, 58, 54 and 61 clones, we found that each of the four neoplastic clones encodes an individual, clonal cellular phenotype, which are shown in Figure 4C,D,E and F respectively.

#### Test-3-specific conclusions

1. The spontaneous evolutions of new individual neoplastic clones from 4-of-4 non-clonal rat hyperplasias with varying aneuploidies within several months in culture is proof-of-principle, that aneuploid hyperplasias are sufficient to generate carcinomas with new clonal karyotypes.
2. Our finding that the four new clonal neoplasms and the two rat carcinomas described above each have individual clonal karyotypes indicates that they each evolved independently by unique karyotypic rearrangements of single cells out of millions of non-clonal, hyperplastic cells with varying aneuploidies. The low probability of such events would explain the individuality of cancer karyotypes.

In following we have asked, What is the mechanism that generates the rare and unique cancer karyotypes from aneuploid hyperplasias?

### Do cancer karyotypes evolve with all clonal aneusomies in single-steps?

To define the question about the origin of cancer karyotypes, we have added up all clonal aneusomies of each of the two carcinomas and the four new clonal neoplasias analyzed here, based on the data summarized in Figures 3, 5, 6, 7, 8, 9 and the accompanying tables: There were *3* clonal aneusomies in RMT 47–3, *5* in RMT 37-2-Clone 1, *8* in RMT-CN 65, *23* in RMT-CN 58, *5* in RMT-CN 54 (relative to normal tetraploidy) and *4* in RMT-CN 61. So the question is, were all 3 to 23 aneusomies of these cancers accumulated gradually or in single-steps? The corresponding gradual and single-step mechanisms make the following testable predictions:

*The gradual mechanism* predicts that karyotypic precursors of cancer cells with sub-cancerous combinations of aneusomies must be stable, because they must survive long enough to allow the accumulation of further cancer-specific aneusomies over subsequent cell generations to become cancer cells. In order to persist during further cell generations, such intermediates would have to be stabilized against destabilizing aneuploidy by selections for some cancer-specific phenotypes, e.g. immortality. The resulting clones of incomplete complements of cancer-specific aneusomies would then accumulate in preneoplastic tissues and would then enhance the probability of carcinogenesis [63]. But there is no evidence for such non-neoplastic clones in the karyotypes of the hyperplasias shown in Tables 2, 3, 4 and 5.

*The single-step mechanism* predicts that all clonal aneusomies of a cancer karyotype have to be acquired at once, because all non-cancerous combinations of aneusomies are unstable (Figure 1). In agreement with this prediction the karyotypes of all carcinomas and neoplastic clones were clonal, whereas the karyotypes of all non-cancerous hyperplasias were non-clonal (Figures 3, 5, 6, 7, 8, 9 and Tables 2, 3, 4 and 5). Accordingly, the non-clonal aneusomies of aneuploid hyperplasias and those spontaneously generated by clonal cancers were never seen twice in a given set of 20 karyotypes (Figures 3, 5, 6, 7, 8, 9) or in consecutive karyotypes of the same culture (not shown here). Prior studies from Heng et al. [64] and from our lab confirm these observations [17,19,24,61,65].

It would follow that the aneuploid cells of non-clonal hyperplasias are too short-lived for the gradual accumulation of cancer-specific aneusomies over many cell generations. In view of this we conclude that cancer karyotypes evolve with all clonal aneusomies in single-steps. This view is supported by independent genetic evidence for “simultaneous gains of chromosomes in a single mitosis” in carcinogenesis, as for example by simultaneous tetraploidization of allelic chromosome pairs [66].

#### Deductions from the single-step mechanism of carcinogenesis

The low odds of forming the new clonogenic karyotype of an autonomous cancer species by the assembly of multiple, inherently unstable aneusomies in a single-step explain the low probability of carcinogenesis and consequently also the long latencies and individualities of cancers.

This single-step mechanism of carcinogenesis also resolves an old paradox of the mutation theory, first pointed out by Rous in 1959 and later by Wolman, that there are very few, if any potential intermediates in carcinogenesis [67,68]. Take the absence of non-cancerous metastases or of immortal but non-cancerous somatic cells as examples.

## Discussion

Here we asked whether the long latent periods from carcinogen to cancers and the individuality of the karyotypes and phenotypes of cancers could be explained by the theory that carcinogenesis is a form of speciation.

### A coherent karyotypic explanation for the long latencies of carcinogenesis and the individuality of cancers

Based on our experiments and the literature on carcinogenesis in rats injected with nitrosourea we found the following chain of events in support of the speciation theory: (1) Nitrosourea induces aneuploidy in rat mammary tissue within 1-day after injection [47]. (2) By unbalancing the karyotype, aneuploidy sets off automatic karyotypic variations. Selections for variants with proliferative phenotypes then generate persistent hyperplasias with varying aneuploidies beginning two months after injection of nitrosourea [44,47,48,52]. (3) Eventually, from about six months after nitrosourea and later rare autonomous cancers with individual clonal karyotypes evolve, which displace the non-clonal and non-autonomous hyperplasias [44,47,48,52]. Since generating a new autonomous cancer cell by random karyotypic variations is inevitably highly inefficient, this mechanism predicts (a) the long latent periods from carcinogens to cancers and (b) the individuality and clonality of cancer karyotypes. We conclude that the speciation theory provides a coherent explanation for the long latencies of carcinogenesis and the individuality of cancers.

The karyotypic cancer theory advanced here derives direct and substantial support from earlier studies by Dulbecco and Armstrong, Aldaz et al. and Goepfert et al. [44,47,48]. These studies described the first evidence for persistent and aneuploid preneoplastic hyperplasias and for clonal karyotypic alterations in cancers in the rat-nitrosourea system. However, a coherent theory about the roles of karyotypes in carcinogenesis did not emerge, because *common* cancer-specific gene-mutations [46,48,69-71] or cancer-specific aneusomies were expected but not found [44,47]. The individual karyotypes and inconsistent mutations (see also next) that were found can, however, now be explained by the speciation theory.

### Single-step origins of cancer-specific karyotypes

The karyotypes of the individual cancers studied here and previously differ from those of corresponding normal cells in multiple numerical and structural chromosome alterations or aneusomies [19,20]. These individual chromosome alterations could have been picked up gradually or in single-steps.

The gradual mechanism of accumulating chromosomal alterations predicts stable precursors in the form of sub-clonal karyotypic intermediates, which we did not observe. Instead, we have found only unstable aneuploid karyotypes in preneoplastic or hyperplastic cells here (Tables 2, 3, 4 and 5) and previously [24,61]. In view of this and the clonal and stable karyotypes of cancers, we concluded that cancer karyotypes must be generated in single-steps, because all non-cancerous aneuploidies are unstable.

Our conclusion is supported by an earlier genetic study of Paulsson et al., who found that the karyotypes of specific leukemias were generated “by simultaneous gain of chromosomes in a single mitosis” [66]. It would follow that carcinogenesis is indeed a sudden or saltational *speciation event* [33,35-37,65,72], as has been postulated for conventional speciation [72-77].

There is, however, a competing theory, which argues that gene mutations are necessary for carcinogenesis, which we discuss briefly.

### Is a gene mutation necessary for nitrosourea-induced carcinogenesis in rats?

Based on a first set of consistent correlations, it has been argued in 1983 that mutations of the cellular H-*ras* gene are necessary for carcinogenesis in rats injected with nitrosourea [46]. But subsequent studies in this system have shown either inconsistent correlations of carcinomas with H-*ras* mutations [47,69,70] or no correlations at all [71]. Moreover, transgenic *ras* genes that were even artificially overexpressed by heterologous promoters proved to be heritable, rather than lethal in mice [78,79]. If tumors developed, those only appeared late in adult mice and, if tested, contained individual clonal karyotypes [18]. Thus proof for a specific oncogenic function of mutant *ras* genes is necessary to confirm a direct role in cancer. But this has not been accomplished yet [80-82].

Our new results, that each rat carcinoma and each neoplastic rat clone has its own individual karyotype and phenotype, now raise new unanswered questions about the role of common mutations, as for example of H-*ras*, in cancers. How would common mutations explain the individualities of cancers?

In view of this we deduce from our current study that the individual phenotypes of the rat carcinomas are encoded by individual karyotypes, independent of mutations, as we have recently shown for the individualities of human cervical carcinomas [49].

## Conclusions

Based on earlier studies of carcinogenesis in the nitrosourea-rat mammary tumor system Dulbecco and Armstrong proposed in 1988, “The similarities between the rat and the human neoplasias suggest that the present findings may be relevant for the evaluation of preneoplastic lesions in breast cancer. If the potential for invasion is also built in human breast cancers from their inception, any evidence pointing to the evolution of a lesion towards malignancy … would have to be considered as an indication of the progressive potential of the lesion” [48]. Our results suggest that the relevant and testable “preneoplastic lesions … to be considered” are the karyotypes of human mammary hyperplasias and cancers.

Our conclusion derives direct support from earlier studies of human breast cancers, finding cancer-specific “nuclear DNA content as an objective biological marker of tumor aggressiveness” [83], and finding that cancer-specific gene expressions are directly proportional to cancer-specific “DNA copy numbers” [84].

## Methods

### Mammary carcinomagenesis in rats injected with a single dose of nitrosourea

The induction of mammary tumors in inbred female rats by single injections of 5 mg of nitrosourea followed long-established procedures [45-48], specifically those described by Aldaz et al. in 1992 [44].

### Cells and cell culture

Rat mammary tumors (RMT) were explanted from rats about 1 year after injection with 5 mg of nitrosourea as described by Aldaz et al. [44]. Explanted tissues were minced with scalpels and dissociated into single cell populations with trypsin. Cells were then propagated in cell culture medium RPMI 1640 (Sigma Co) supplemented with 5% or 10% fetal calf serum, 1% Anti-Anti (GibCo Company) and 1% Nystatin (Sigma Company).

### Karyotype analysis

One to two days before karyotyping, cells were seeded at about 50% confluence in a 5-cm culture dish with 3 ml medium containing 5% fetal calf serum. After reaching ~75% confluence, 250 ng colcemid in 25 μl solution (KaryoMax, Gibco) was added to 3 ml medium. The culture was then incubated at 37°C for 4–8 hrs. Subsequently cells were dissociated with trypsin, washed once in 3 ml of physiological saline and then incubated in 0.075 molar KCl at 37°C for 15 min. The cell suspension was then cooled in ice-water, mixed (‘prefixed’) with 0.1 volume of the freshly mixed glacial acetic acid-methanol (1:3, vol. per vol.) and centrifuged at 800 g for 6 minutes at room temperature. The cell pellet was suspended in about 100 μl supernatant and than mixed drop-wise with 5 ml of the ice-cold acetic acid methanol solution and incubated at room temperature for 15 min. This cell suspension was then pelleted once more as above and re-suspended in a small volume of the acetic acid-methanol solution. An appropriate aliquot was transferred with a micropipette tip to a glass microscope slide and allowed to evaporate at room temperature. Slides with suitable metaphase chromosome spreads were hybridized with chromosome-specific-color-coded DNA probes as described by the manufacturer (MetaSystems, Newton, MA 02458). Karyotypes were then prepared as described by us previously [17,49].

Note: All work on animals has been approved by the Animal Care and Use Committee of the University of California at Berkeley.
